# Dual-energy CT in musculoskeletal imaging: technical considerations and clinical applications

**DOI:** 10.1007/s11547-024-01827-6

**Published:** 2024-05-14

**Authors:** Domenico Albano, Filippo Di Luca, Tommaso D’Angelo, Christian Booz, Federico Midiri, Salvatore Gitto, Stefano Fusco, Francesca Serpi, Carmelo Messina, Luca Maria Sconfienza

**Affiliations:** 1 IRCCS Istituto Ortopedico Galeazzi, Milan, Italy; 2 Dipartimento di Scienze Biomediche, Chirurgiche ed Odontoiatriche, Università degli Studi di Milano, Milan, Italy; 3 Scuola di Specializzazione in Radiodiagnostica, Università degli Studi di Milano, Milan, Italy; 4 Diagnostic and Interventional Radiology Unit, BIOMORF Department, University Hospital Messina, Messina, Italy; 5 Department of Radiology and Nuclear Medicine, Erasmus MC, Rotterdam, The Netherlands; 6 Division of Experimental Imaging, Department of Diagnostic and Interventional Radiology, University Hospital Frankfurt, Frankfurt am Main, Germany; 7 Dipartimento di Scienze Biomediche per la Salute, Università degli Studi di Milano, Milan, Italy

**Keywords:** Dual-energy CT, Musculoskeletal, Gout, Bone marrow edema, Metal artifact reduction

## Abstract

Dual-energy CT stands out as a robust and innovative imaging modality, which has shown impressive advancements and increasing applications in musculoskeletal imaging. It allows to obtain detailed images with novel insights that were once the exclusive prerogative of magnetic resonance imaging. Attenuation data obtained by using different energy spectra enable to provide unique information about tissue characterization in addition to the well-established strengths of CT in the evaluation of bony structures. To understand clearly the potential of this imaging modality, radiologists must be aware of the technical complexity of this imaging tool, the different ways to acquire images and the several algorithms that can be applied in daily clinical practice and for research. Concerning musculoskeletal imaging, dual-energy CT has gained more and more space for evaluating crystal arthropathy, bone marrow edema, and soft tissue structures, including tendons and ligaments. This article aims to analyze and discuss the role of dual-energy CT in musculoskeletal imaging, exploring technical aspects, applications and clinical implications and possible perspectives of this technique.

## Introduction

The introduction of dual-energy CT (DECT) technology has started a new phase in CT imaging characterized by precision and versatility. The pioneers and theorists of DECT were initially Godfrey Hounsfield in 1973 and then Alvarez and Macovski in 1976, who described, respectively, how two different images could be acquired in the same slice at 100 kV and 140 kV and how from the polychromatic X-ray spectrum it would be possible to obtain energy-dependent information by separating the measured attenuation coefficients [1–3]. This intuition then evolved into the elaboration and subsequent development of modern CT platforms with dual energy sources.

The focal and most innovative point of DECT is precisely the use of two energy spectra of X-rays whom elaboration allows obtaining essential clinical information in several settings [4–6].

A relevant possible application of DECT is oncology, in which the advantages are represented by a better characterization of tumor tissues. This potential lies in the capability of DECT to differentiate between healthy and diseased parenchyma [7]. Through the reconstruction of iodinated maps DECT permits to visualize the vascularization of the neoplasm in a more reliable approach and therefore allowing a more adequate characterization of the lesions after chemotherapy or antiangiogenic therapy [8–12]. Similarly, in cardiovascular imaging, it has been demonstrated that DECT permits a reliable assessment of coronary pathology through the detection of coronary plaques, myocardial perfusion defects and myocardial scar tissue [13–16], all crucial elements in risk stratification and patient management.

DECT has spread like wildfire also due to the limits of its direct competitor, single-energy CT (SECT). Indeed, SECT is based exclusively on a single energy spectrum of X-rays. When tissues show overlapping attenuation values, SECT may struggle to provide the clear differentiation needed for a precise diagnosis [17]. This may lead to diagnostic challenges when attempting to characterize focal lesions, evaluate vascular conditions, or identify materials within the body. Additionally, SECT is more prone to artifacts, streaking, and beam dispersion, which are common problems that can compromise image quality of SECT [18]. Over the last years, an increasing application of DECT is musculoskeletal imaging [19, 20]. This paper is aimed to analyze the current and major applications of DECT in musculoskeletal disorders analyzing technical aspects and possible perspectives of this imaging modality.

## Technical considerations

DECT involves the use of both low and high energy spectra to decompose the material [21]. Usually, the two energy spectra are around 80–90 kV and 140–150 kV, respectively. The principles underlying DECT are mainly represented by the photoelectric effect, which is related to the X-ray beam energy and the atomic number of tissues, and Compton effects, which depends on tissue electronic density. The combined information from these two effects retrieved by applying low and high energy levels allows to improve the understanding of tissues properties.

The acquisition of DECT images has been performed in various ways and is classically divided as source- or detector-based. The former can be distinguished in dual-source DECT, fast kilovolt switching, and sequential acquisition. Dual source DECT involves the use of two tubes and related detectors set at different kV (typically 80 and 140) with the concurrent activation of both tubes [22]. The fast kilovolt switching approach consists of just one rotation of the tube that rapidly changes voltage in a very short time (< 0.2 ms), switching from low to high kVp values [22, 23]. The sequential acquisition approach is among the first methods introduced in DECT technology, which consists in the acquisition of data at low-kVp and a subsequent acquisition at high-kVp values [24]. So different methods of images acquisition can be based on DECT source. For what concerns detector-based acquisition we find dual-layer detector (DLCT) and photon-counting detectors (PCCD). DLCT is designed as a conventional single energy source (tube voltage ranging from 100 to 140 kVp) and a layered detector. The inner and outer layers consist of zinc selenide or cesium iodide crystals and gadolinium oxysulfide, respectively, absorbing low-energy and high-energy photons [25]. PCCD are the latest advancement of currently available CT technology and may represent the new frontier for CT imaging, being capable of assessing more than two different energy levels. These detectors made by semiconductors materials allow converting the radiation energy into electrical signal, resulting in a significant reduction of electronic noise and the possibility to better discriminate different energies by application of specific thresholds [26]. PCCD may allow to overcome some limitations of standard CT like by removing electronic noise, increasing spatial resolution, small low-contrast structures detection and image quality in contrast-enhanced examinations, also providing useful spectral information. Among the potential challenges of PCCD the cost is certainly non-negligible as well as the urge of highly performing algorithms and software that will be able to process the huge amount of data provided by this imaging modality.

## DECT algorithms

**Fig. 1:**
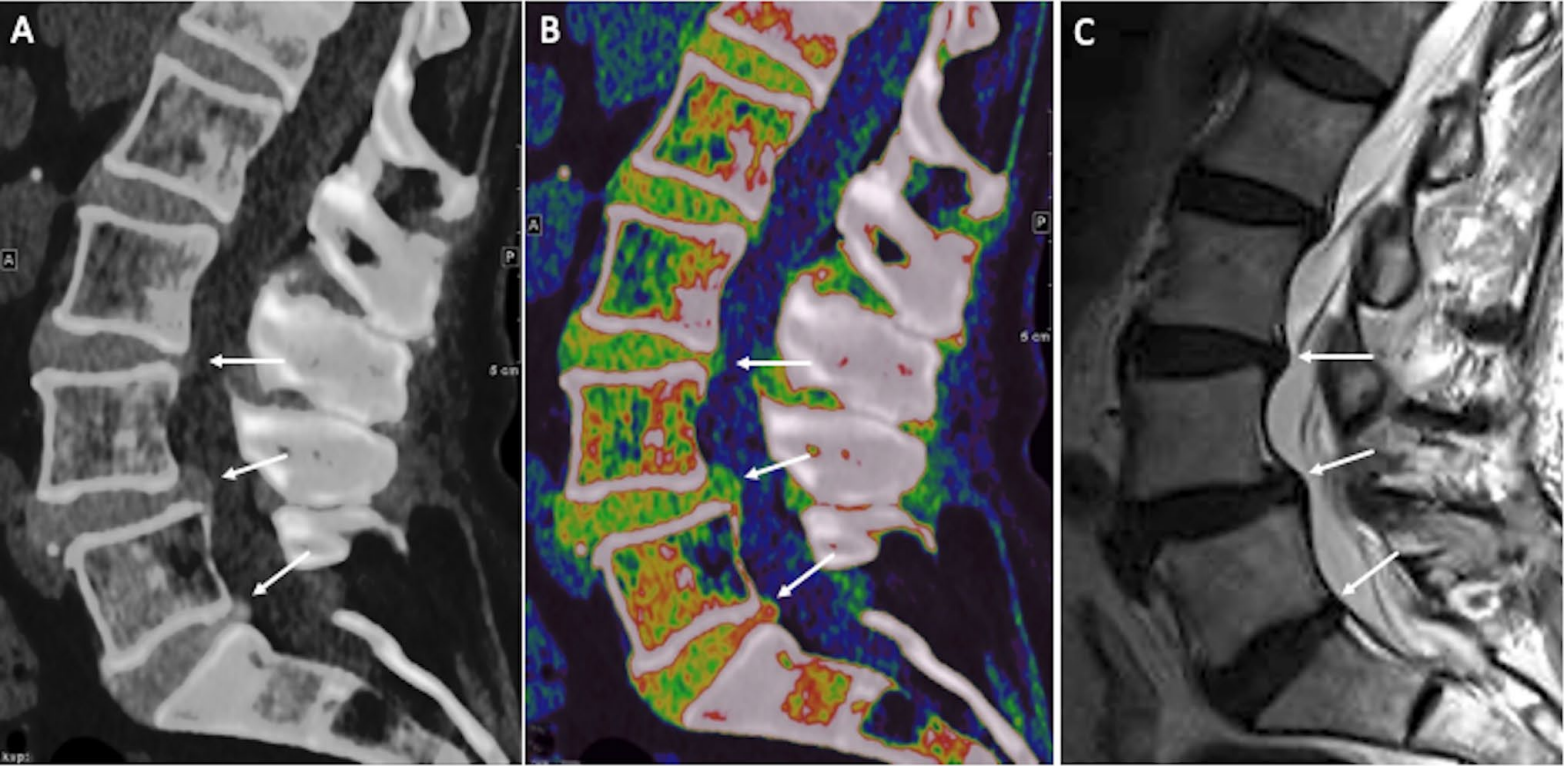
Lumbar spine images of a 58-year-old man with low back pain. Standard sagittal unenhanced CT **a** show L4/L5 degenerative spondylolisthesis, with L3/L4 and L4/L5 lumbar disk protrusions and L5/S1 disk herniation (arrows). Disk protrusions and herniation are better depicted on sagittal VNCa DECT image (**b**) and sagittal T2-weighted MRI image (**c**)

A panel of images is produced from the different energy sources. Higher soft tissue contrast is provided by the low energy set with lower noise using the high energy set. In DECT, dual-energy data acquisition is just the starting point of the imaging process, followed by post-processing data to extract valuable information and to improve diagnostic capabilities. A series of DECT algorithms can be applied and might be helpful for musculoskeletal imaging. Notably, virtual non-contrast (VNC) imaging is a post-processing method utilized to create "unenhanced" images from through the subtraction of iodine. Indeed, the iodinated contrast can be removed, making the acquisition of a basal phase unnecessary, thus reducing radiation exposure [27]. Iodine quantification is another specific application enabled by post-processing. DECT may enable to quantify the concentration of iodine in tissues, offering information on blood perfusion and identifying areas of increased iodine absorption, as observed in tumors [28]. Color-coded Virtual Noncalcium (VNCa) images have proven to be reliable for the visualization of bone marrow edema (BME) and soft tissue structures (Fig. 1). These reconstructions have also enhanced the identification of bone marrow infiltration by tumors. A further feature of DECT in post-processing is the creation of virtual monoenergetic images (VMI) at several energy levels. The VMI concept relies on the variation of materials attenuation with X-ray energy. By combining information from both energy levels, VMI can be generated at a specific virtual energy level [29]. Last, DECT images quality is further enhanced in post-processing phase reducing artifacts and ensuring that the final images are of the highest diagnostic quality, for instance, decreasing metal-related artifacts, image registration to reduce motion artifacts, filters for noise reduction [30].

## Musculoskeletal applications

In musculoskeletal imaging, DECT may have a pivotal role in different clinical scenarios. Some of them are already consolidated applications of this imaging modality.

### Metal artifact reduction technique

Metal artifact reduction is a crucial point for musculoskeletal radiologists who routinely deal with patients presenting metal implants [31]. In this setting, metal-related artifacts can make challenging detection and evaluation of periprosthetic fractures, infection, pseudotumor, implant failure, dislocation, and rupture [32]. Traditionally, CT images experience quality deterioration because of heightened beam attenuation from metal implants. Currently, metal artifact reduction (MAR) techniques have been applied, for instance, iterative reconstruction (IR) algorithms. MAR algorithms select the corrupted data due to metal element, consequently replacing these data with interpolated data of neighboring detectors [33]. MAR approach is highly effective when huge dealing with huge amount of metal, but secondary artifacts may be generated. DECT offers new possibilities by using two different energy levels to reduce image degradation. VMIs are acquired through two different energy levels using DECT. The beam with higher energy undergoes reduced attenuation and consequently less beam hardening, whereas the lower energy beam delivers superior contrast for soft tissues, with an optimal balance between 105 and 130 kV [34, 35]. Lee et al. investigated the capability of decreasing artifacts of DECT by studying 40 subjects with metallic implants using post-processing with monoenergetic data obtained at 70 keV and 150 keV; images were compared to conventional CT performed on 40 controls matched [36]. Conventional CT presented much lower (*p* < 0.001) values within the fat (− 301 HU vs. − 115 HU) and muscles (− 405 HU vs. − 96 HU) covering the metal, substantial lower signal-to-noise ratio and quality of images compared to High-kV DECT reconstruction, even with slightly higher radiation dose (about 14 mGy and 19 mGy, respectively, *p* = 0.08) [36, 37]. Donders investigated 41 subjects with suspected nonunion surgically treated with internal fixation through positioning of metallic implants [38]. Low-kV DECT showed significant (*p* < 0.001) lower images quality and confidence of the readers than high-kV DECT, also with higher rate of false-negatives (*p* = 0.283). Barreto et al. performed a comparison of image quality of six cadavers with bony metallic implants examined through MAR algorithm, conventional CT, and DECT [39]. In all scans, MAR was favored over traditional CT, whereas traditional CT was favored over DECT. Due to the diminished soft tissue contrast and persistent artifacts with DECT, assessing surrounding structures posed challenges. In the case of the cervical spine, DECT was the only scenario where it lessened the intensity of metal artifacts and enhanced the clarity of all structures. According to their conclusions, MAR proved to be more efficient in minimizing metal artifacts compared to DECT. Nevertheless, it was noted that different artifacts with slight streaking appeared on MAR images. In cases where the implant is relatively compact, DECT might outperform MAR without introducing different artifacts [39]. Also, it seems that the amalgamation of VMI and MAR yielded the most significant artifacts [37]. Indeed, VMI is particularly useful for evaluating the metal–bone interface and for reducing hyperdense artifacts, with the iterative MAR approach being able to improve the assessment of soft tissues reducing hypodense artifacts. Of note, there is no standardized optimal VMI energy level to decrease metal-related artifacts, given that it depends on the metal alloy, shape and geometry of the metal components.

### Gout imaging—urate detection

**Fig. 2:**
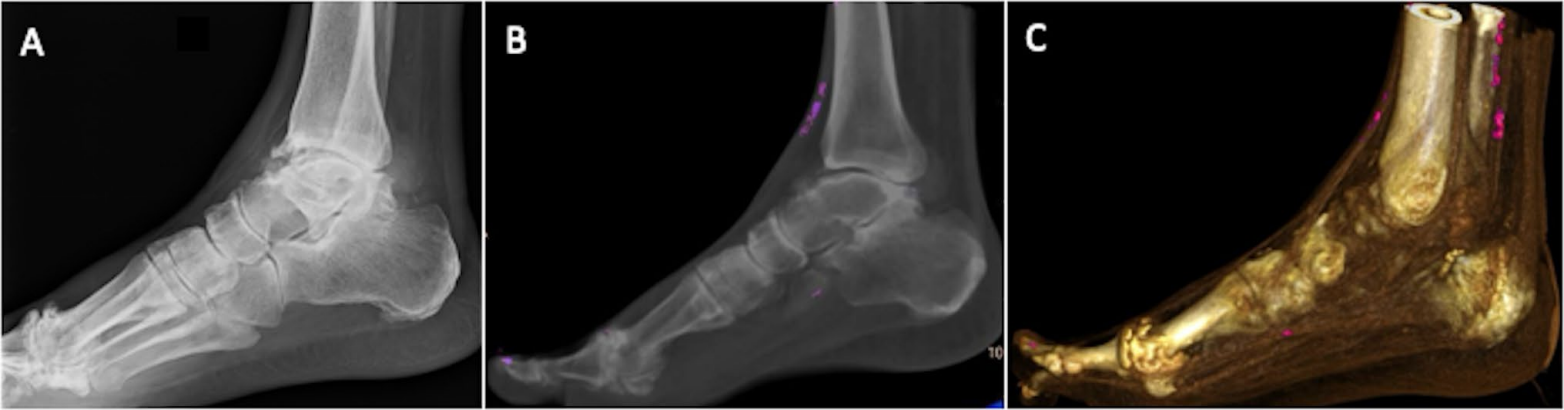
Acute ankle pain in 72-year-old male patient with gout. Lateral X-ray view **a** shows osteoarthritis of the tibio-talar and first metatarsophalangeal joints. Post‐processed sagittal average intensity projection (**b**) and volume-rendered (**c**) color-coded DECT images show urate deposits colored in purple

DECT has been documented to exhibit higher sensitivity (up to 89%) and specificity (up to 100%) to reach the diagnosis of gout when compared with other imaging modalities [40–43]. The use of DECT is opening the possibilities of new diagnostic scenarios by identifying, mapping, and monitoring crystal deposits [43]. DECT takes advantage of the different Dual energy index (DEI) of calcium and urate. Once these materials have been differentiated, each can be uniquely color-coded (Fig. 2). As a matter of fact, the technological advancements of DECT and validation of dedicated gout-tools resulted in a robust imaging modality that has been included in the 2015 Gout classification criteria [44]. Furthermore, the accuracy of DECT for gout diagnosis can be substantially different in different phases of the disease. In fact, while it reaches accuracy of 100% in active phase of chronic gout with arthritis and tophus, it results positive in 36–80% of patients with acute symptomatic gout flare (< 6 weeks) and in 62–100% of symptomatic patients with intercritical gout (less than 3 years) [45]. Of note, radiologists must be aware of possible artifacts when utilizing DECT gout protocols. One of the main studies on artifacts related to the study of gout in DECT was conducted by Mallinson et al. who analyzed DECT of 50 subjects with presumed gout in order to stratify the most frequent types of artifacts evaluating multiple joints [46]. Artifacts were observed in 90% of scans including:

*Nail bed artifacts* these artifacts can be observed in the nails of the feet (88% of subjects) with less frequency in the hands (4 % of subjects). The most plausible explanation was the similarity of DEI values of keratin and monosodium urate.

*Skin artifacts* these artifacts, again, were common in the feet (about 40% of cases) but less in the hands (about 4% of cases). No skin artifacts were found on the knees or elbows. One reason may be the greater amount of calloused skin in the foot. However, these artifacts were observed old scanners images, having become negligible over time in the most recent machines.

*Submillimeter artifacts* believed to be a form of noise. However, it is necessary to be aware of their veracity and to consider them as gouty deposits if they are found in an anatomical structure such as in tendons. Again, this issue has been overcome in the most recent scanners.

Anyway, despite these artifacts can be encountered in DECT gout protocols, they are readily recognized, so false-positive findings quite rare. A further artifact was found in the study corresponding to the calcific vessels. Three out of four patients with this type of artifact showed further similar artifacts at the level of the arteries of the lower limbs, suggesting a possible meaning of gouty deposit, the statistical significance of which, however, could not be demonstrated due to the small sample. Indeed, it has been postulated that urate might contribute to the dysfunction of vascular endothelium [47].

Some limitations of DECT in gout imaging must be pointed out. In addition to the abovementioned artifacts, time-consuming post-processing, and non-negligible radiation exposure, DECT is not able to detect crystal deposits smaller than 2 mm located over the cartilage or within the effusion [48]. Then, it is less sensitive than MRI and ultrasound in detecting synovitis, tenosynovitis, and BME [49, 50]. Last, its accuracy decreases in deeper joints (i.e., spine or hip) due to the poor penetration of low voltage beam.

### Calcium pyrophosphate dehydrate disease (CPPD)

Pseudogout is an arthropathy presenting with the deposition of calcium pyrophosphate dehydrate (CPPD) crystals in joints and neighboring tissues. This condition, also named pseudogout, may be suspected when chondrocalcinosis is identified on X-ray images, and arthrocentesis may aid in confirming the diagnosis. The diagnosis of pseudogout can be reached when joint and neighbor soft tissues mineral deposits without MSU are detected by DECT. This imaging modality proved to be valuable in pseudogout given that it is able to depict and quantify CPPD crystals. DECT has shown 78% sensitivity in detecting CPPD deposits, surpassing that of X-ray (44%) [51]. Although DECT may not be the optimal choice for early identification of CPPD deposits, it is believed to offer higher diagnostic performance than conventional CT [52].

### Bone marrow edema detection

**Fig. 3:**
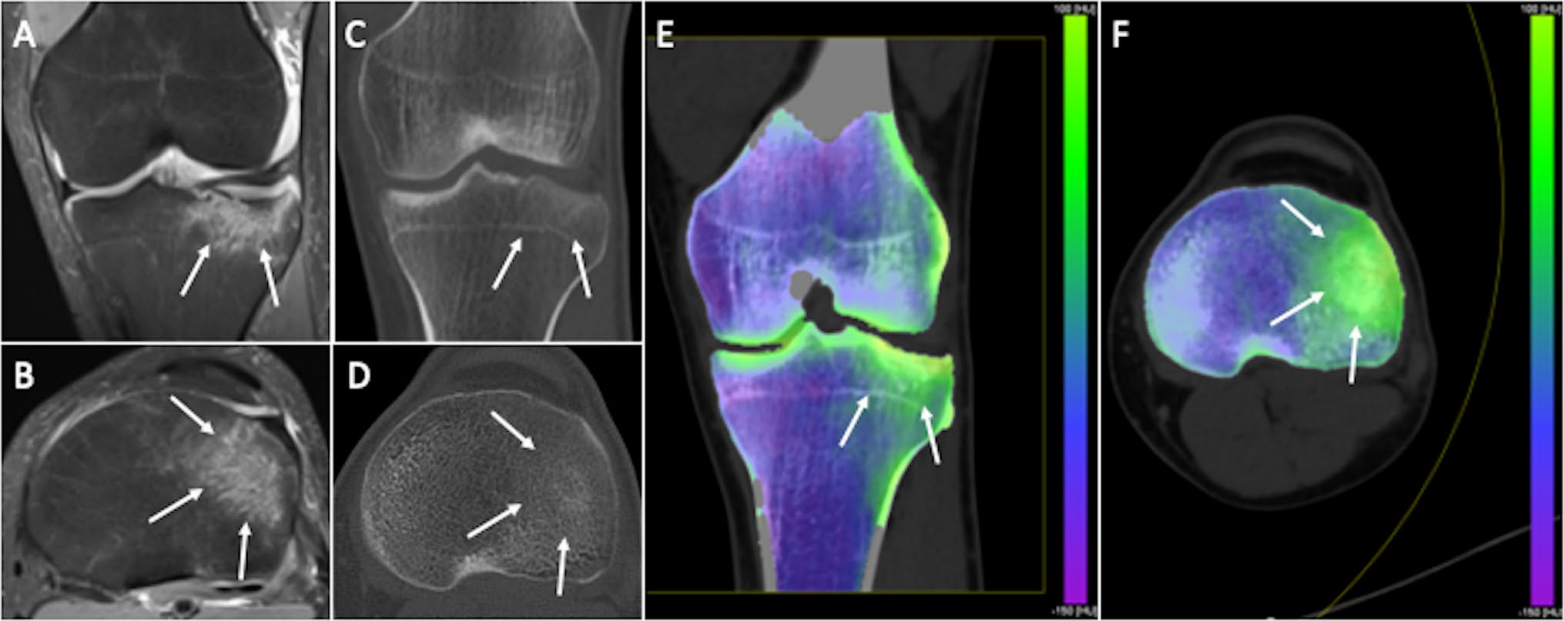
Traumatic tibial fracture in a 32-year-old woman. Coronal (**a**) and axial (**b**) fat-suppressed proton density weighted images show diffuse bone edema of lateral tibial plateau (arrows). Coronal reformat (**c**) and axial (**d**) standard CT images seem to show bone impaction of lateral tibial plateau as subtle subchondral hyperdensity (arrows), while the corresponding coronal (**e**) and axial (**f**) virtual noncalcium DECT images clearly demonstrate bone edema (arrows)

Conventional CT remains inadequate to identify BME, which has always been exclusive prerogative of MRI [53–58]. VNCa DECT algorithm allows to eliminate the contribution of calcium to attenuation values of the bone [59, 60]. Notably, VNCa imaging may have some struggles in detecting subcortical changes making challenging the identification of subchondral edema due to osteochondral lesions [61]. Cavallaro et al. in a study on the column of 88 patients have highlighted the advantages of DECT over MRI in the context of post-traumatic BME. DECT has been shown to have the ability to identify BME that is comparable to MRI with the advantage of being able to make the fracture line more visible (Fig. 3). DECT has been shown to be highly accurate for depicting BME presence and amount, with about 85–90% sensitivity and 98% specificity, also with significantly higher diagnostic confidence when compared with MRI (*p* < 0.001). Furthermore, a threshold of -0.43 Hounsfield yielded a 89% sensitivity and 90% specificity of 90% in identifying BME, resulting in an overall 0.96 area under the receiver operating characteristic curve [62].

DECT has widely been used to diagnose BME mostly in traumatic settings, but some studies have also proven the applicability of DECT on the depiction of BME in non-traumatic subjects. Chen et al. recently published a meta-analysis and review including ten papers including a total of 2463 anatomic regions in non-traumatic patients, yielding overall 88.4% sensitivity, 96.1% specificity, and 0.98 area under the receiver operating characteristic curve for the detection of BME [63]. In a study conducted on the lower limbs of 44 patients suspected of having osteomyelitis, Foti et. al reported about 90% sensitivity and 80% specificity values of DECT in the detection of BME [64]. Furthermore, in a study of 40 subjects with presumed sacroiliitis, Chen et al. obtained about 77% sensitivity and 90% specificity [65]. The diagnostic performance in the identification of BME in post-traumatic and non-traumatic musculoskeletal pathology of the main studies is in line. DECT presents itself as an excellent radiological diagnostic alternative in those musculoskeletal clinical conditions predisposing to BME: avascular necrosis, infection, stress fractures and bone tumors [63].

As abovementioned for deeper anatomic structures, also BME detection may be challenging in larger patients. In these cases, 100 kVp should be applied for the lower-energy acquisition when a dual-source system is available, while patients over than 120–125 kg should not be investigated with single-source systems [66]. Then, all processes altering the attenuation of the bone marrow, like normal red marrow or marrow hyperplasia with phenomenon of marrow conversion, might be misinterpreted as BME. Hence, training is essential for a correct interpretation of BME in DECT and comparison with the contralateral side (i.e., for pelvis and limbs) may be helpful.

### Ligaments and tendons

**Fig. 4:**
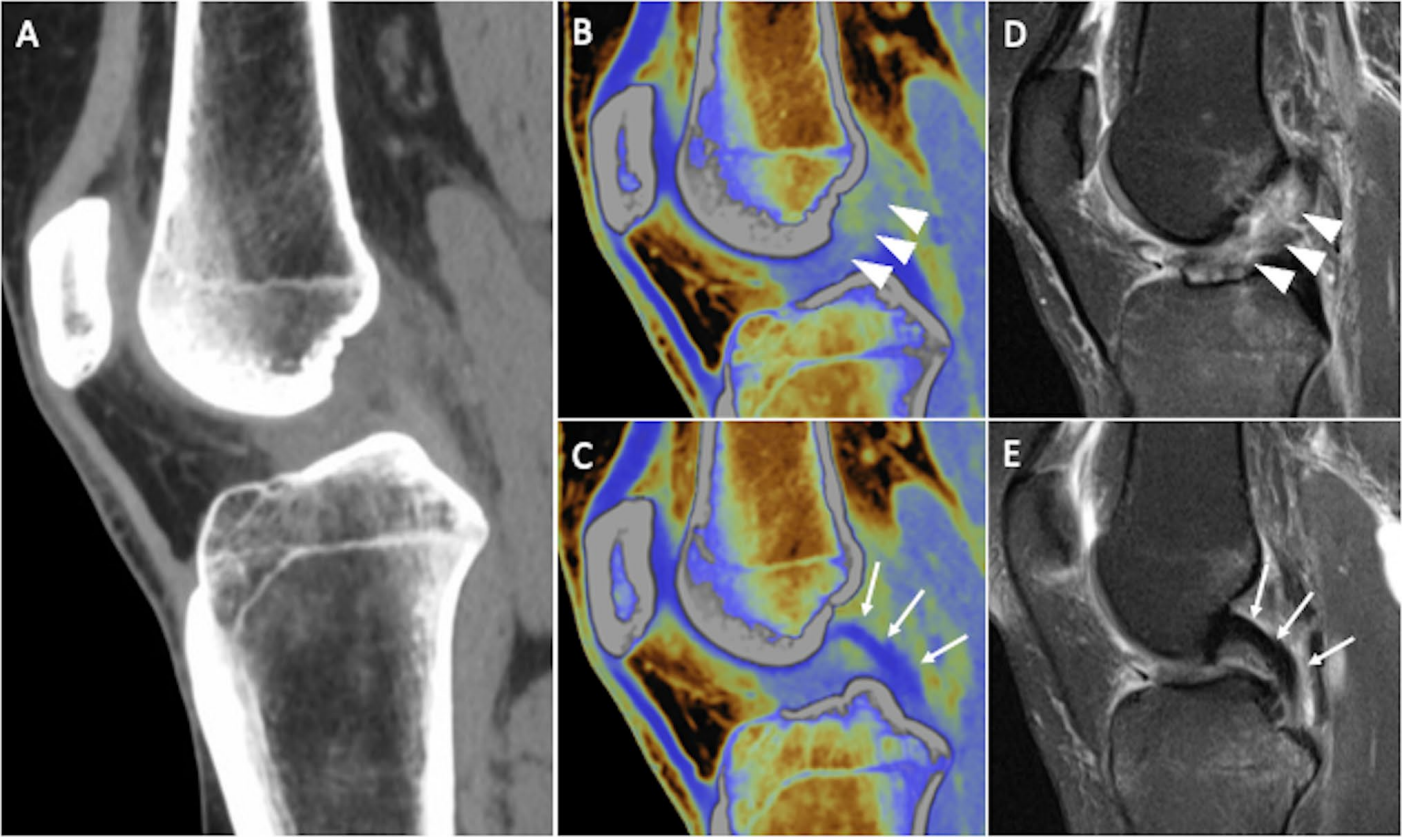
Anterior cruciate ligament tear in a 42-year-old man after knee sprain. Sagittal reformat of standard knee CT (**a**) is not helpful for describing clearly cruciate ligaments status. Sagittal virtual noncalcium DECT images allow the identification of anterior cruciate ligament injury (**b**, arrowheads), with normal appearance of posterior cruciate ligament (**c**, arrows), as proven by the corresponding sagittal fat-suppressed proton density weighted MRI images (**d** and **e**, respectively)

Ultrasound and MRI are well-established modalities to image soft tissue structures [67–71]. Nevertheless, DECT can be used as a complementary imaging modality. DECT can be employed to distinguish collagenous structures in soft tissues based on their comparatively high density and DUAL-energy indices (DEIs) by using a specific algorithm for tissue decomposition. So, tendons and ligaments can be identified and then color-coded to be integrated with gray-scale images, aiding in the identification of pathological changes [19]. DECT has proven to be an interesting alternative technique for the visualization of tendons and ligaments, with promising data concerning its capability to detect soft tissues injuries, especially in the emergency setting [72]. Liu et al. investigated 51 subjects and 102 knees, focusing on anterior cruciate ligament, has strengthened the literature which highlights how in acute cases, where MRI has some limitations in the evaluation of ligament structures, DECT represents a valid opportunity. Overall, DECT had excellent performance in detecting anterior cruciate ligament status with sensitivity, specificity, positive predictive value, negative predictive value, and accuracy of 97–98%, with no different accuracy when compared with MRI (*p* > 0.99) (Fig. 4) [73]. The authors used MRI and arthroscopy as reference for evaluating the diagnostic performance of DECT.

### Detection of bone lesions

Despite other imaging modalities have been traditionally used for this purpose [74–78], different ways may be used to detect bone metastases or locations of multiple myeloma through DECT. Standard CT is generally applied to identify metastatic lesions or bony localizations of hematological malignancies. CT has limited sensitivity for bone lesions, particularly when they present similar density of normal bone. DECT may help to discriminate the attenuation values of normal bone from those of malignancies at different energy spectra given that metastases and hematological proliferative disorders show different tissue compositions with respect to normal bone marrow. DECT can identify these subtle differences using quantitative tissue decomposition, which involves mathematical algorithms and analysis techniques that use the acquired DECT data to calculate the proportions or concentrations of different materials within a given voxel or region of interest. These algorithms are able to estimate the relative amounts of different components. In metastases, neoplastic tissues infiltrate the bone marrow with increased water content related to the high vascular permeability of malignancies [79]. Malignant lesions often exhibit heterogeneous compositions with varying densities and vascularity. DECT can identify areas of necrosis, hemorrhage, and calcification within the lesion, which are more commonly associated with malignancy. Benign lesions, on the other hand, tend to have more uniform compositions without significant necrosis or vascularity [80, 81]. As a matter of fact, DECT increases tumor conspicuity, especially of focal bone isodense lesions, that might result in improved tumor detection in CT scans. It would be particularly worthful in follow-up of cancer patients. Furthermore, quantitative analysis of material decomposition images may provide valuable insights into changes in lesion composition, volume, or vascularity, aiding in the assessment of responses to therapy. Differently, qualitative tissue decomposition can be used in DECT to better depict cortical and trabecular bone, even subtracting these tissues thereby making tumors more evident. In a previous study, sensitivity of CT for bone metastases increased from 76–80% to 87–93% by using DECT-derived color maps [82]. In this regard, a potential pitfall could be related to cortical lesions, given the limit of DECT in removing calcium components, but these tumors are generally easily recognized on standard CT. Another pitfall could be the misinterpretation of focal red marrow hyperplasia or atypical hemangiomas that may decrease the specificity of DECT. Then, VNC imaging and iodine mapping can be helpful tool for the identification of iodine containing lesions by iodine subtraction. The better iodine enhancement observed by iodine density maps in high density structures like bones is indeed an advantage of DECT for the identification of enhancing tumors [83]. Hence, VNC imaging may improve the sensitivity of CT in depicting bone metastases [84]. Iodine mapping and attenuation quantification have been shown to be helpful in a retrospective study including about 700 bone metastases on chest DECT done on 54 subjects, in which DECT found more than 90% of skeletal metastases confirmed on technetium-99 bone scan [85]. Another interesting point is the opportunity to differentiate peri-skeletal tissues extension from reactive edema by highlighting the different iodine concentration and slope curve [86]. Qualitative analysis of DECT images on VNCa images has shown to increase the diagnostic performance of DECT if compared to monoenergetic CT [83]. Bone metastases and myeloma present as hyperattenuating lesions on VNCa. A drawback of VNCa images in myeloma is that locations of disease can be hardly differentiated from reactivated marrow, with VNCa being more accurate in bone with poor red marrow.

## Conclusion

In summary, DECT stands out as a robust and adaptable imaging technique, demonstrating notable progress in musculoskeletal imaging. Its capacity to generate high-quality images with similar radiation exposure to CT positions it as a robust resource for radiologists, particularly as a complementary imaging modality in acute scenarios and oncologic settings in addition to MRI. Within the musculoskeletal system, DECT is highly regarded to image crystal arthropathies, BME identification, and mitigating artifacts related to metallic implants. As technology advances and new techniques emerge, it is reasonable that DECT will be a more and more indispensable imaging modality for radiologists.
